# Tannins from *Hamamelis virginiana* Bark Extract: Characterization and Improvement of the Antiviral Efficacy against Influenza A Virus and Human Papillomavirus

**DOI:** 10.1371/journal.pone.0088062

**Published:** 2014-01-31

**Authors:** Linda L. Theisen, Clemens A. J. Erdelmeier, Gilles A. Spoden, Fatima Boukhallouk, Aurélie Sausy, Luise Florin, Claude P. Muller

**Affiliations:** 1 Institute of Immunology, Centre de Recherche Public de la Santé/Laboratoire National de Santé, Luxembourg, Luxembourg; 2 Department of Preclinical Research, Dr. Willmar Schwabe GmbH & Co. KG, Karlsruhe, Germany; 3 Department of Medical Microbiology and Hygiene, University Medical Centre of the Johannes Gutenberg University, Mainz, Germany; German Primate Center, Germany

## Abstract

Antiviral activity has been demonstrated for different tannin-rich plant extracts. Since tannins of different classes and molecular weights are often found together in plant extracts and may differ in their antiviral activity, we have compared the effect against influenza A virus (IAV) of *Hamamelis virginiana* L. bark extract, fractions enriched in tannins of different molecular weights and individual tannins of defined structures, including pseudotannins. We demonstrate antiviral activity of the bark extract against different IAV strains, including the recently emerged H7N9, and show for the first time that a tannin-rich extract inhibits human papillomavirus (HPV) type 16 infection. As the best performing antiviral candidate, we identified a highly potent fraction against both IAV and HPV, enriched in high molecular weight condensed tannins by ultrafiltration, a simple, reproducible and easily upscalable method. This ultrafiltration concentrate and the bark extract inhibited early and, to a minor extent, later steps in the IAV life cycle and tannin-dependently inhibited HPV attachment. We observed interesting mechanistic differences between tannin structures: High molecular weight tannin containing extracts and tannic acid (1702 g/mol) inhibited both IAV receptor binding and neuraminidase activity. In contrast, low molecular weight compounds (<500 g/mol) such as gallic acid, epigallocatechin gallate or hamamelitannin inhibited neuraminidase but not hemagglutination. Average molecular weight of the compounds seemed to positively correlate with receptor binding (but not neuraminidase) inhibition. In general, neuraminidase inhibition seemed to contribute little to the antiviral activity. Importantly, antiviral use of the ultrafiltration fraction enriched in high molecular weight condensed tannins and, to a lesser extent, the unfractionated bark extract was preferable over individual isolated compounds. These results are of interest for developing and improving plant-based antivirals.

## Introduction

Human influenza A viruses (IAV) cause seasonal epidemics, with three to five million cases and 250,000–500,000 deaths worldwide every year [1]. While vaccination is safe and effective in preventing infections, current vaccines require annual reformulations to account for the antigenic drift of new IAV strains. In addition, it takes months between the emergence of a new potentially pandemic strain and the availability of the vaccine. Although during the 2012 influenza season more than 98% of the tested H1N1 strains were sensitive to oseltamivir and zanamivir [2], resistance to antivirals [3]–[5] has been reported, e.g. from the UK [6] and Australia [7]. Therefore, the continuous development and improvement of antivirals is an important public health priority.

HPVs are non-enveloped DNA viruses whose low-risk subtypes can cause genital warts, while high risk subtypes (e.g. HPV 16 or 18) can be at the origin of ano-genital malignancies such as cervical carcinoma. Since 2006, two effective vaccines against HPV are licensed, but they protect only against a minor fraction of the over 100 serotypes. Also, high costs may limit their use especially in developing countries. Protection from HPV by the use of condoms has been a matter of debate [8]–[12]. An alternative approach is to prevent HPV infection by developing formulations for topical application (e.g. in lubricants), which was successfully demonstrated with carrageenan, a linear sulfated polysaccharide [13], [14] and with polyanionic or polycationic molecules [15]–[17]. In addition, recurrence of genital warts after treatments such as cryotherapy or surgery is high (about 30%, [18], [19]), because lesions in the surrounding tissue provide a new access for HPV particles to basal cells. Topical application of a drug inhibiting HPV infection could lower recurrence after these interventions. It is therefore of interest to identify new compounds that inhibit HPV infection.

Antimicrobial activity has been demonstrated for many plant extracts; active compounds often belong to the classes of terpenoids, alkaloids, lectins or polypeptides, but mostly to the phenolics [20]. An important group of antimicrobial phenolics are the tannins. Tannins are secondary plant metabolites defined by their ability to precipitate proteins, a property usually inherent to tannins with a molecular weight from 500–3000 g/mol [21]. Their binding affinity and ability to precipitate proteins depends, in addition to the tannińs molecular weight, also on protein size and structure, as well as on reaction conditions (pH, temperature, solvent, time) [22]–[24]. Soluble or insoluble complexes can be reversibly formed [23], [25]. Tannins are multidentate ligands, binding to proteins mainly by hydrophobic interactions and hydrogen bonds [23], [26], [27]. In addition to this rather unspecific binding, also highly specific binding, for example of epigallocatechin gallate (EGCG) to the HIV glycoprotein 120 binding pocket of the CD4 T-cell receptor has been demonstrated [28].

**Figure 1:**
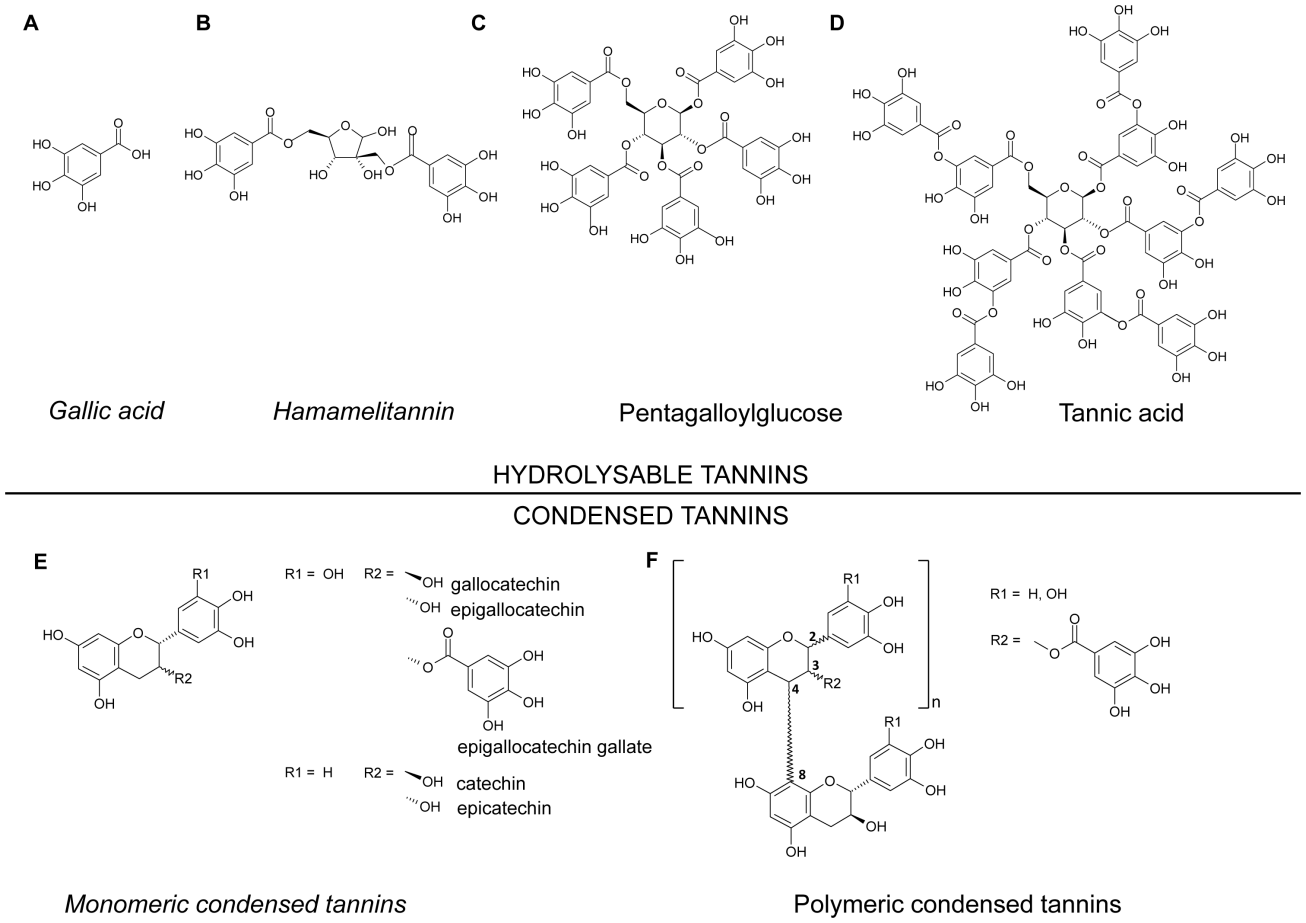
Chemical structures of tannins and pseudotannins in *Hamamelis virginiana* L. (**A**) gallic acid, (**B**) hamamelitannin, (**C**) pentagalloylglucose, (**D**) tannic acid represented with 10 galloylation units, (**E**) monomeric condensed tannins, (**F**) polymeric condensed tannins [48]. Pseudotannins having low or no protein precipitating activity (and molecular weights <500 g/mol) are shown in italics.

Tannins from higher plants are subdivided into two classes: hydrolysable tannins and non-hydrolysable or condensed tannins (also known as proanthocyanidins [23], [29]). Hydrolysable tannins are based on gallic or ellagic acid moieties, while condensed tannins are based on flavan structures. In this study, we focused on both hydrolysable and condensed tannins as well as their low molecular weight (<500 g/mol) non-precipitating moieties such as gallic acid, which are also referred to as pseudotannins (see also Fig. 1).

Tannin-rich plant extracts have shown antimicrobial effects. For example EPs® 7630, an extract from *Pelargonium sidoides*, prevented attachment of group A streptococci to epithelial cells [30] as well as IAV infection *in vitro* and *in vivo* [31]. Anti-infectious properties have also been demonstrated for isolated tannins. EGCG for instance, was active against *Streptococcus pyogenes* [32] and staphylococci [33] as well as against hepatitis C virus, HIV or IAV [34]–[36]. Gallic and tannic acid inhibited IAV growth in embryonated eggs [37], pentagalloylglucose influenced IAV infectivity and release [38].

Antiviral activity has been demonstrated for selected tannins. However, different classes and molecular weights of tannins are often found together in plant extracts, and may differ in their antiviral activities. Nevertheless, there are only few systematic comparisons of their anti-IAV structure-activity relations. For condensed tannins, we have previously shown that the anti-IAV effect increases with their polymeric chain length [31], and the importance of the 3-galloyl group was shown for monomeric catechins [36]. A better understanding of the antiviral activity of different tannin categories and structures against IAV and HPV is warranted to optimize plant-based antivirals in view of higher selectivity indices.

To investigate differential antiviral activities of tannins, we chose *Hamamelis virginiana* L. (Hamamelidaceae) extracts as model extracts. This shrub-like deciduous tree originates from the Eastern part of North America. Pharmaceutical extracts or distillates are primarily obtained from the bark or leaves. Due to their antiphlogistic and astringent properties, these extracts are widely used in skin care, to treat small wounds, local inflammations [39]–[41], or hemorrhoids [42]. In addition, antimutagenic as well as antioxidant properties have been described [43]–[45].

Hamamelis bark extract is an ideal candidate to investigate differential antiviral activities because it is rich in tannins, which account for as much as 8–12% of the bark weight [46], and its tannins and pseudotannins are diverse and well characterized (Fig. 1). Ethanolic bark extract contains about 31% of condensed tannins [47], which are mainly composed of (epi)catechin and (epi)gallocatechin moieties, linked preferably by 48 interflavan bonds [48]. Up to 29-mers have been detected in the extract and while the terminal catechin units are not galloylated, chain extender units are completely galloylated at position 3 [48]. In addition to condensed tannins, Hamamelis bark contains various hydrolysable tannins and pseudotannins. Besides the major compound hamamelitannin, gallic acid as well as carbohydrates with up to 10 galloyl moieties, such as pentagalloylglucose (5 galloylations) or tannic acid (≤10 galloylations), have been identified [49]–[52].

Antiviral activity of Hamamelis extracts has so far been demonstrated only against herpes simplex virus [47]. We report here for the first time on the efficacy of Hamamelis extracts against IAV and HPV. We compared the antiviral effect against IAV of bark and leaf extracts, fractions enriched in tannins of different molecular weights and individual tannins of defined structures, including pseudotannins. We investigate the anti-IAV structure-activity relations of (pseudo)tannins, cytotoxic effects and antiviral mechanisms, highlighting differences between tannins from different classes and molecular weights. We identified and characterized a highly potent fraction inhibiting early life cycle steps of both IAV and HPV. This fraction was obtained by enrichment of high molecular weight condensed tannins using ultrafiltration, a simple, reproducible and easily upscalable method.

## Materials and Methods

### Plant Extracts, Fractions and Isolated Compounds

Full extracts (60% ethanol) of *Hamamelis virginiana* L. leaf or bark, as well as ultrafiltration (UF) concentrates and filtrates were prepared as previously described [47]. For bark extract fractionation into UF-concentrate and filtrate, a Pro Flux M12 Tangential Flow Filtration System (Millipore) with Pellicon 2 Ultrafiltration Cassettes (C Screen, Millipore) was used. All extracts and UF-fractions were prepared at Dr. Willmar Schwabe GmbH & Co. KG, Karlsruhe and provided as dry powders. Concentrations indicated designate dry extract weight per volume of solvent. Gallic acid monohydrate (“gallic acid”), penta-O-galloyl-β-D-glucose (“pentagalloylglucose”), hamamelitannin, tannic acid and (−)-epigallocatechin gallate (EGCG) were purchased from Sigma-Aldrich. The buffer capacity of the cell culture medium fully neutralized the acidity of compounds added to *in vitro* experiments.

### Cell Culture

Madin-Darby canine kidney (MDCK, American Type Culture Collection) cells were cultivated in EMEM containing 10% fetal bovine serum, 25 mM HEPES, 2 mg/ml bovine serum albumin and antibiotics. DMEM supplemented with 10% fetal bovine serum, 2 mM ultraglutamine and antibiotics was used for A549 (American Type Culture Collection), A549Slam [53] and VeroSlam [54] cells (both supplied by Y. Yanaga, Fukuoka, Japan). A549Slam and VeroSlam stably express the Slam receptor essential for certain measles strains. Non-virally transformed keratinocytes (HaCaT, Cell Lines Services) were grown in DMEM supplemented with 10% fetal bovine serum, 2 mM GlutaMAX I (Invitrogen), 1% modified Eagle’s medium nonessential amino acids and antibiotics. Cell culture reagents were purchased from Lonza unless otherwise indicated.

### Virus Stock Culture and Titrations

IAV stocks (H1N1 A/Puerto Rico/8/34, pandemic H1N1 A/Luxembourg/46/2009, seasonal H3N2 A/Luxembourg/01/2005, H7N9 A/Anhui/01/2013) were grown on MDCK cells using serum free virus growth medium supplemented with 2 µg/ml L-1-tosylamido-2-phenylethyl chloromethylketone-(TPCK) trypsin (Sigma-Aldrich). Half maximal tissue culture infectious dose (TCID50) determinations of IAV were done on MDCK cells, incubating them in quadruplicates for 3 days at 37°C and 5% CO2 with 3-fold serial dilutions of virus-containing supernatant. The cytopathic effect was scored and TCID50 was calculated by the ID-50 5.0 program (http://www.ncbi.nlm.nih.gov/CBBresearch/Spouge/html_ncbi/html/index/software.html#1). Adenovirus (Type 5, ATCC reference strain) was propagated and titered by TCID50 determination on A549 cells, measles virus stocks (Schwarz vaccine strain, GSK, Belgium) on VeroSlam cells.

### Selectivity Index (SI) Determination

Cytotoxicity was assessed in 96-well plates (Greiner BioOne) with 3⋅10^4^ A549 cells per well incubated for 24 h with 2-fold serial drug dilutions using the Cell proliferation kit II (XTT, Roche Diagnostics) according to the manufacturer’s instructions. This kit is based on the conversion of XTT to an orange formazan salt by metabolically active cells. The antiviral effect against a GFP reporter virus (H1N1 A/Puerto Rico/8/34-NS116-GFP) [55] was determined in serum free A549 medium containing 0.2 µg/ml TPCK-trypsin by applying a 2-fold drug dilution series on triplicates of 3⋅10^4^ A549 cells per well directly after addition of the reporter virus. A multiplicity of infection (MOI) of 0.4 was used for optimal fluorescent readout of the virus batch. Fluorescence was read after 24 h at 535 nm (excitation at 485 nm) on a Tecan Genios Plus Reader (Tecan GmbH, Austria). The half maximal cytotoxic concentration (CC50) and the half maximal antiviral concentration (EC50) were determined by SigmaPlot 12 from 2 to 3 independent experiments (carried out in at least triplicates). The selectivity index was calculated as SI = CC50/EC50.

### Antiviral Efficacy Testing against Different IAV Wild Type Strains, Adenovirus, Measles and HPV

IAV infection of A549 cells was performed in serum free A549 medium containing 0.2 µg/ml TPCK-trypsin. A549 cells were infected with a MOI of 0.1 of wild type IAV unless mentioned otherwise, or with a MOI of 0.05 for adenovirus type 5. A549Slam cells were infected with a MOI of 0.01 of measles virus. The antiviral drugs were added immediately after the virus unless stated otherwise. After 24 h (48 h for measles; 24 h, 48 h and 72 h for A/Puerto Rico/8/34), the supernatant was centrifuged and the TCID50 was determined as described under Section 2.3. Time of addition studies as well as virus and cell preincubation experiments were performed similarly, with the modifications described in the corresponding Result Section. HPV type 16 pseudoviruses were prepared and used for infection as previously described [17], [56], [57]. Briefly, they were produced by cotransfection of codon-optimized HPV L1 and L2 cDNA and a pcDNA3.1/luciferase reporter plasmid into 293TT cells [56]. Virions were purified by Optiprep gradient centrifugation. Thousand pseudovirions per HaCaT cell were added 1 h after the drug dilutions and luminescence was read after 24 h using Luciferase Assay System (Promega) and expressed in percent of untreated control. Experiments were done in at least triplicates.

### Preparation of Tannin-free Extracts and Quantification of Condensed Tannins and Phenols

Tannins were depleted from extract or single compound solutions in phosphate buffered saline (PBS) under continuous stirring with 25 mg/ml (antiviral efficacy experiments) or 50 mg/ml (hemagglutination/neuraminidase assay) of hide powder (FILK, Freiberg) for 1 h at room temperature. In this process, tannins bind to the hide proteins, precipitate and can be removed by filtration (Whatman cellulose filters grade 1). Phenolics, the main constituting moieties of both hydrolysable and condensed tannins, were quantified before and after hide powder treatment by Folin-Ciocalteu’s phenol reagent. This is the standard method of the European Pharmacopoeia [58], [59] for quantification of total phenolics based on their reducing capacities. Briefly, 2 volumes of pyrogallol standard (Sigma-Aldrich) or sample, 1 volume Folin-Ciocalteu reagent (Sigma-Aldrich), 10 volumes of water were mixed and 12 volumes Na_2_CO_3_ (290 g/L, Sigma-Aldrich) were added. After 30 minutes of incubation at room temperature, absorbance was read at 760 nm on a SpectraMax Plus plate reader (Molecular devices). Phenol content was determined using a pyrogallol standard curve, expressed as pyrogallol equivalents (PGE) and PGE of hide powder treated samples was normalized to PGE of untreated samples, corresponding to 100%. To estimate reproducibility of the extract and UF-fraction preparation, the amount of only condensed tannins was determined using the acid-butanol method [60] as we used in a previous publication [47]. Therefore, the drugs were heated for 2 h at 95°C with 5% concentrated hydrochloric acid in n-butanol and absorbance was measured at 550 nm.

### Hemagglutination and Neuraminidase Inhibition Assays

For the hemagglutination inhibition assay, 20 µl of drug serial dilutions or PBS were mixed with 30 µl of the lowest H1N1 A/Luxembourg/46/2009 concentration still agglutinating erythrocytes (2.4×10^5^ TCID50) or PBS in round-bottom wells. 50 µl of a 0.75% washed human erythrocyte solution in PBS was added and hemagglutination was scored after 60 min. The half maximal hemagglutination inhibiting concentration (HIC50) was calculated using ID-50 5.0.

For the neuraminidase inhibition assay, 50 µl of drug serial dilutions or PBS were added to 2.4×10^5^ TCID50 of A/Luxembourg/46/2009 H1N1 in MES (2-(N-morpholino)ethanesulfonic acid)-based assay buffer or to 50 µl virus-free assay buffer in a 96 well black µClear plate and incubated for 45 min. 50 µl of 0.3 mM 2′-(4-methylumbelliferyl)-α-D-N-acetylneuraminic acid (Sigma-Aldrich) was added followed by 1 h incubation at 37°C and addition of stop solution (0.166 M NaOH in ethanol). Fluorescence (ex 360 nm, em 448 nm) was read on an Infinite M200 plate reader (Tecan) and background fluorescence (without virus) was subtracted. The half maximal neuraminidase inhibiting concentration (NIC50) was calculated using SigmaPlot 12. Both assays were run in triplicate and in up to three independent experiments.

### Drug Cytotoxicity, Apoptosis Induction and Unspecific Effects on Host Cell Receptors

Metabolic activity of A549 or HaCaT cells was determined in triplicates using the Cell proliferation kit II (XTT, Roche Diagnostics) 24 h after adding the compounds. Caspase 3/7 activity of A549 cells after 24 h of incubation with the drugs, with 2.5 µM staurosporine (Enzo Life Sciences) or with DMSO was measured using Caspase-Glo 3/7 Assay (Promega). The assay is based on cleavage of a substrate by caspase 3 or 7 to luminogenic aminoluciferin.

Interference of the drugs with cellular TNF-α signaling was investigated as previously described [61]. Briefly, 30 ng/ml TNF-α were added to A549 cells 30 minutes before or at the same time than drug treatment. Total proteins were extracted 15 minutes later using CHAPS buffer and IκB-α was detected by Western blot using a rabbit anti-IκB-α antibody (C-21, Santa Cruz). As a loading control, β-actin was detected using a mouse anti-β-actin antibody (Santa Cruz). Cy-5 and Cy-3 labelled appropriate secondary antibodies were used (GE Healthcare) and fluorescence was detected on a Typhoon TRIO+ scanner (GE Healthcare).

### HPV Binding and Capsid Disassembly Assay

For the binding assay, HaCaT cells were preincubated for 1 h with the original or tannin-free (Section 2.6) extracts or DMSO and were then infected for 15 min with 500 HPV pseudovirions per cell. Cells were washed five times with PBS and collected in SDS sample buffer for Western blotting. Cell-bound HPV16 particles were stained with anti-L1 antibody 312F [62]. β-Actin (loading control) was stained using a murine antibody (Sigma-Aldrich) and relative band intensities were quantified densitometrically.

For the HPV capsid disassembly assay, HaCaT cells were grown on coverslips and treated with 20 µg/ml of the extracts for 1 h before HPV16 pseudovirion infection for 7 h at 37°C. Cells were fixed with methanol and stained with mouse anti-L1 antibody (33L1-7) as described previously [63], [64]. L1-7 recognizes an epitope located inside of the pseudovirion capsid and is only accessible after uncoating [65]. Fluorescence was recorded using a Zeiss Axiovert 200 M microscope. For quantification, the relative amount of internalized particles was determined based on the L1-7-positive pixels relative to the cell nucleus signal (DNA/Hoechst 33342-positive pixels) out of 100 randomly selected cells from two independent experiments. A threshold value was set to exclude background.

### Statistical Methods

Data are presented as mean ± standard deviation. Statistical analyses were done in SigmaPlot 12 (Systat Software) using Mann-Whitney Rank Sum test or Pearson correlation. p<0.05 was considered as significant.

## Results

### Antiviral Activity of Hamamelis Bark and Leaf Full Extract

**Figure 2:**
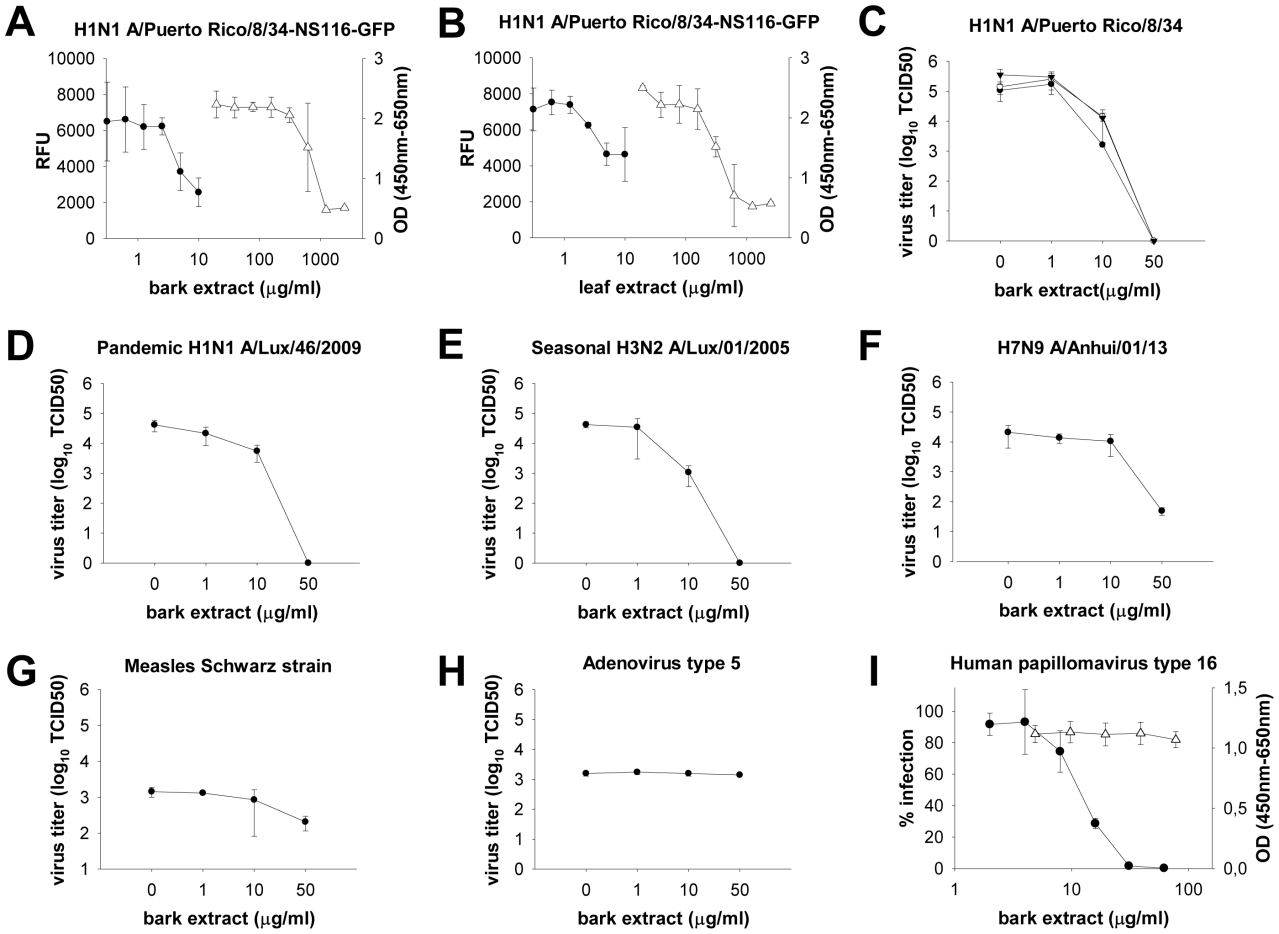
Antiviral activity of Hamamelis bark extract. (**A–B**) Selectivity index determination. Fluorescence of an H1N1 reporter virus A/Puerto Rico/8/34-NS116-GFP (MOI 0.4 on A549 cells) after 24 h of treatment with Hamamelis bark (**A**) or leaf (**B**) extract, expressed in relative fluorescent units (RFU, closed circles). Cytotoxicity of bark (**A**) or leaf (**B**) extract on A549 cells after 24 h as determined by XTT assay (open triangles). Background absorbance at 650 nm has been subtracted from XTT absorbance at 450 nm. A representative of at least two independent experiments is shown. (**C–H**) Antiviral activity of the bark extract against wild type strains. A549 cells (or A549Slam for measles) were infected in triplicates with an MOI of 0.1 for H1N1 A/Puerto Rico/8/34 (**C**), pandemic H1N1 A/Lux/46/2009 (**D**), seasonal H3N2 A/Lux/01/2005 (**E**), H7N9 A/Anhui/01/2013 (**F**), a MOI of 0.01 for measles Schwarz strain/Rimevax (**G**) or a MOI of 0.05 for adenovirus type 5 ATCC reference strain (**H**) in presence of Hamamelis bark serial dilutions. TCID50 was determined after 24 h (**C–F, H** closed circles), 48 h (**C**, open circles, **G**), or 72 h (**C**, closed triangles). (**I**) Activity of bark extract against 1000 HPV 16 pseudovirions per HaCaT cell in triplicates. Luminescence was read after 24 h and expressed in percent of untreated controls (closed circles). Cytotoxicity of bark extract on HaCaT cells was determined after 24 h by XTT assay (open triangles) as described for panel 2A. OD, optical density.

Hamamelis bark and leaf full extracts were tested for their antiviral activity against IAV and their cytotoxic effect by XTT assay on A549 cells. Both had approximately the same antiviral efficacy against the H1N1 strain A/Puerto Rico/8/34-NS116-GFP (EC50 = 5.2 or 3.9 µg/ml respectively), but the bark extract showed a lower cytotoxicity. Therefore, the bark extract had a SI of 94.7 compared to 57.1 for the leaf extract (Fig. 2AB, Table 1) and was chosen for further investigation.

**Table 1:** Cytotoxic and antiviral activities against an H1N1 reporter virus of Hamamelis extracts and fractions.

	CC50, µg/ml	EC50, µg/ml	SI (CC50/EC50)	Enriched in
**Bark full extract**	495.1	5.2	94.7	/
**Leaf full extract**	223.6	3.9	57.1	/
**UF-concentrate**	349.3	1.1	325.5	≥ tetrameric CT
**UF-filtrate**	968.9	36.2	26.7	HT,<tetrameric CT

Half maximal cytotoxic concentration (CC50), half maximal antiviral concentration (EC50) against A/Puerto Rico/8/34-NS116-GFP after 24 h of treatment with serial dilutions of antiviral compounds (Section 2.4.) Selectivity index SI = CC50/EC50. CT, condensed tannins, HT, hydrolysable tannins.

The bark extract showed a dose-dependent reduction in titers on all IAV strains tested. Viral growth was completely abolished at 24 h post infection at ≥50 µg/ml for the H1N1 laboratory strain A/Puerto Rico/8/34 (Fig. 2C), the currently circulating pandemic H1N1 (Fig. 2D) and seasonal H3N2 strains (Fig. 2E) and was reduced >400-fold for the recently emerged avian H7N9 IAV (Fig. 2F). The antiviral effect persisted at 48 and 72 h post infection (Fig. 2C). At the same concentrations, the bark extract had no substantial effect on measles (Schwarz strain, Fig. 2G) or type 5 adenovirus (ATCC reference strain, Fig. 2H). Concentrations ≥31 µg/ml of bark extract reduced relative infection of the HPV 16 pseudoviruses below 2% compared to the untreated control cultures, in absence of cytotoxicity on HaCaT cells in the XTT assay (Fig. 2I).

### Antiviral Structure-activity Relations of Hydrolysable Tannins and Pseudotannins

Tannins are major constituents of Hamamelis bark and the antiviral potential of tannin-rich extracts or single tannins has been described [38], [66]–[68]. However, a direct systematic comparison of the anti-IAV effects of hydrolysable tannins and pseudotannins is of interest. After 24 h of incubation with H1N1 A/Puerto Rico/8/34-NS116-GFP, the EC50s of gallic acid, pentagalloylglucose (5 galloylations) and tannic acid (≤10 galloylations, see Fig. 1 for structures) were determined as 50.8 µM, 19.5 µM and 4.3 µM respectively. Thus, the anti-IAV effect increased with the number of galloylations for these compounds on a molar basis (Table 2). Hamamelitannin did not show any anti-IAV activity up to 10 mM (Table 2). With CC50s of 770.5 µM for gallic acid, 779.4 µM for pentagalloylglucose and 132 µM for tannic acid, SIs of 15.2, 40.0 and 30.7 were determined (Table 2). EGCG, a monomeric condensed tannin carrying one galloylation was chosen for comparison to hydrolysable tannins and showed a higher SI (85.0) than any other single (pseudo)tannin (Table 2). Comparison to a polymeric condensed tannin was not possible due to the unavailability of an isolated, well defined high molecular weight compound. Interestingly, the bark full extract showed a higher SI (94.7, Table 1) than any of the single compounds (Table 2).

**Table 2:** Cytotoxic and antiviral activities against an H1N1 reporter virus of hydrolysable tannins and pseudotannins.

	CC50		EC50		SI		Gall.	Mmol	
	µM	*(µg/ml)*	µM	*(µg/ml)*					g/mol
**Hamamelitannin**			>10 mM						
**Gallic acid**	770.5	*(144.9)*	50.8	*(9.6)*	**15.2**	1			188.1
**Pentagalloylglucose**	779.4	*(733.1)*	19.5	*(18.3)*	**40.0**	5			940.7
**Tannic acid**	132.0	*(224.4)*	4.3	*(7.3)*	**30.7**	≤10			1701.2
**EGCG**	1029.1	*(471.8)*	12.1	*(5.6)*	**85.0**	1			458.4

Half maximal cytotoxic concentration (CC50), half maximal antiviral concentration (EC50) against A/Puerto Rico/8/34-NS116-GFP after 24 h of treatment with serial dilutions of antiviral compounds (Section 2.4). Selectivity index SI = CC50/EC50. Gall., number of galloylations; Mmol, molecular weight. Molar mass of tannic acid calculated as carrying 10 galloylations.

### Antiviral Activity of Hamamelis Bark Extract Enriched in High Molecular Weight Tannins by Ultrafiltration

**Figure 3:**
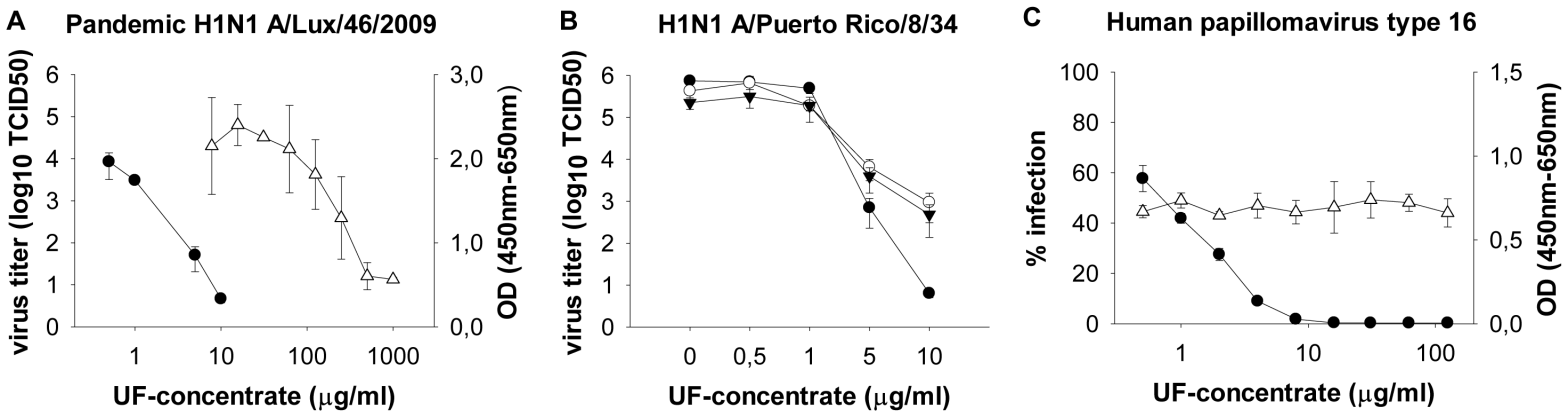
Antiviral activity of UF-concentrate, a fraction of the bark extract enriched in high molecular weight tannins. (**A–B**) A549 cells were infected in triplicates at a MOI of 0.1 with pandemic H1N1 A/Lux/46/2009 (**A**) or H1N1 A/Puerto Rico/8/34 (**B**) and serial dilutions of UF-concentrate were added at the same time. TCID50 was determined at 24 h (**A, B**, closed circles), 48 h (**B**, open circles) or 72 h (**B**, closed triangles) post infection. (**C**) Activity of bark extract against 1000 HPV 16 pseudovirions per HaCaT cell, done in triplicates. Luminescence was read after 24 h and expressed in percent of untreated control (closed circles). (**A, C**) Cytotoxicity of UF-concentrate on A549 (**A**, open triangles) or HaCaT cells (**C,** open triangles) was determined after 24 h by XTT assay. Background absorbance at 650 nm has been subtracted from XTT absorbance at 450 nm. OD, optical density.

In order to remove the antivirally inactive hamamelitannin (Table 1, [47]) and because it has been shown that the effect of condensed tannins increases with molecular weight [31], the bark extract was fractionated by ultrafiltration (UF) through a 3 kDa membrane. In a previous publication [47], the acid butanol method [60] was used for condensed tannin quantification in similar UF-fractions. Using the same method, we determined the overall condensed tannin content as 33.2% (bark extract), 66.2% (UF-concentrate) and 17.1% (UF-filtrate). The comparison with the previously published contents (30.9%, 62.3%, 14.6%, respectively, [47] shows good reproducibility of the extraction and fractionation procedure. The UF-filtrate (<3 kDa) was shown to be enriched in low molecular weight tannins (monomers, dimers, trimers) and the UF-concentrate (≥3 kDa) in tetrameric and longer condensed tannins [47]. Importantly, UF-concentration nearly doubled the condensed tannin content and increased the SI from 94.7 for the full extract to 325.5 for the UF-concentrate (Table 1), which corresponded to the highest SI of all compounds tested. In contrast, the SI of the UF-filtrate (fraction <3 kDa) decreased by more than three-fold to 26.7 (Table 1). The high anti-IAV activity of the UF-concentrate was confirmed on wild type IAV strains: after 24 h of treatment, 10 µg/ml of UF-concentrate reduced viral titers of pandemic H1N1 as well as of H1N1 A/Puerto Rico/8/34 strains by >3 or >5 logs, respectively, on A549 cells (Fig. 3AB, closed circles), while 50 µg/ml of bark extract were needed to achieve comparable titer reductions (Fig. 2CD). Also the anti-HPV 16 effect was enhanced, as infection of the pseudoviruses dropped below 2% compared to the untreated controls from ≥8 µg/ml of UF-concentrate (Fig. 3C, closed circles) as compared to ≥31 µg/ml for bark extract. While in parallel to a 4.7-fold increase in anti-IAV efficacy, also a 1.4-fold increase in cytotoxicity was observed on A549 cells for the UF-concentrate as compared to the bark full extract (Table 1, Fig. 2A, Fig. 3A, open triangles), there was no cytotoxicity detectable by XTT assay at antiviral concentrations for both A549 and HaCaT cells (Fig. 3AC, open triangles). Thus, for Hamamelis bark extract, concentration of high molecular weight tannins by ultrafiltration is a convenient and reproducible method to increase the antiviral SI.

### Determination of the Active Antiviral Principle in Hamamelis Extracts

**Figure 4:**
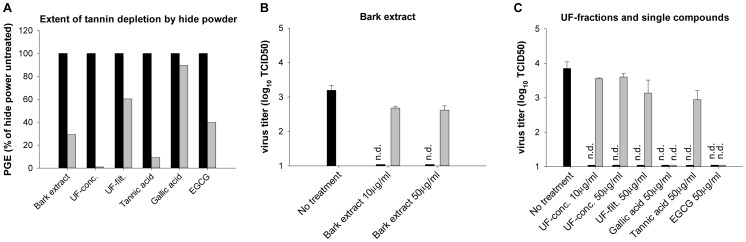
Determination of the active antiviral principle in Hamamelis bark extract. (**A**) Extent of tannin depletion by precipitation with hide powder. Tannins were depleted from drug solutions by stirring with hide powder for 1 h at room temperature followed by filtration. Phenolics, the main constituting moieties of tannins, were photometrically quantified before (black bars) and after (grey bars) hide powder treatment by Folin-Ciocalteu’s phenol reagent using a pyrogallol standard curve. Pyrogallol equivalents (PGE) of hide powder treated samples were normalized to PGE of untreated samples, set to 100%. (**B–C**) Antiviral effect of tannins. A549 cells were infected in triplicates with pandemic H1N1 A/Lux/46/2009 (MOI 0.05) and were left untreated or treated for 24 h with bark extract (**B**), UF-fractions or isolated (pseudo)tannins (**C**) which had been (grey bars) or had not been (black bars) treated with hide powder. Titers were determined at 24 h post infection by TCID50. n.d., not detectable or TCID50<1.

Previous results from our group [31] and the comparison of the Hamamelis extracts and UF-fractions have shown that the anti-IAV effect increases in parallel to the molecular weight of their condensed tannins. Similarly, the binding efficiency of tannins to proteins increases with their molecular size [26]. To see if (i) tannins are the antiviral principle of the Hamamelis extracts and (ii) tanning (protein precipitating) activity is needed for antiviral efficacy, we removed tannins from the bark extract using hide powder [59]. By incubation of a drug solution with hide powder, compounds with tanning activity bind to the collagen in the hide powder, precipitate, and can be removed by filtration. In general, polyphenols with molecular weight from 500–3000 g/mol usually precipitate proteins [21]. Therefore, monomeric catechins or gallic acid (<500 g/mol) can normally not or only incompletely be removed from plant extracts by hide powder. Phenols, the main constituting moieties of both hydrolysable and condensed tannins, were quantified before and after hide powder treatment by Folin-Ciocalteu’s phenol reagent [58], [59]. Tannins were efficiently removed (remaining phenol content <1% or <10% of untreated) from the long molecular weight tannin containing UF-concentrate and tannic acid, but not from gallic acid or the UF-filtrate rich in low molecular weight constituents (89% or 60% remained, Fig. 4A). Phenols in bark extract, containing both high and low molecular weight tannins, and EGCG showed intermediate reduction (Fig. 4A). In the anti-IAV assay, ≥10 µg/ml of the bark full extract completely abolished growth of pandemic H1N1 (MOI 0.05) after 24 h of incubation, but even 50 µg/ml of tannin-depleted extract did not have a similar effect (Fig. 4B). Also for the UF-fractions and the single compounds, the antiviral effect was abolished after successful tannin removal, but not if large amounts of low molecular weight polyphenols remained in solution (gallic acid, EGCG, Fig. 4C). Thus, tannins do mediate the antiviral effect, while the tanning activity *per se* is not absolutely required, as can be seen by the remaining antiviral effect of tannin-free gallic acid and EGCG.

### Determination of the Affected Step of the Viral Life Cycle

**Figure 5:**
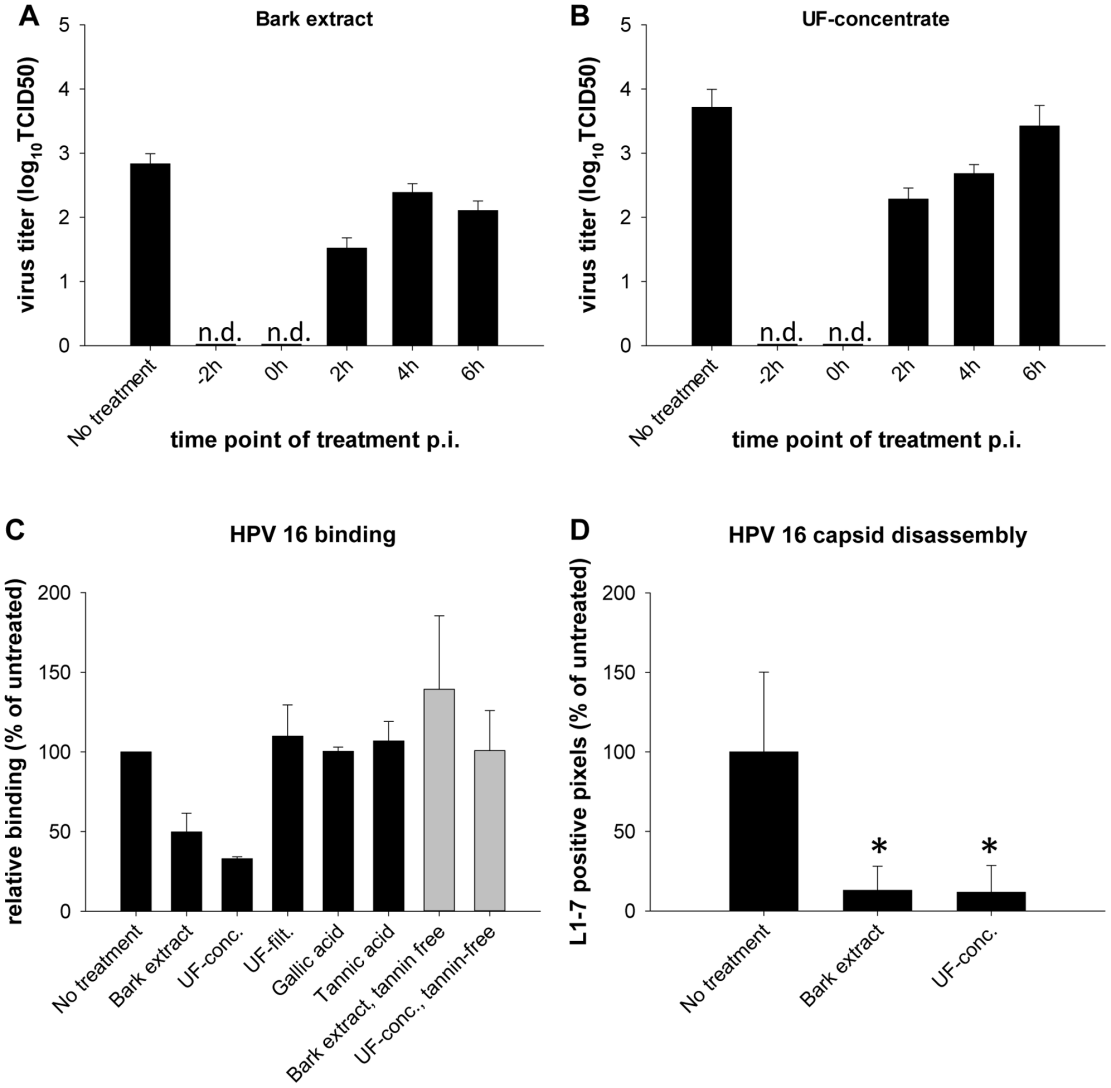
Effect on different IAV and HPV life cycle steps. (**A–B**) Step of the IAV life cycle affected. A549 cells were infected with pandemic H1N1 (MOI 0.1), and treated with 50 µg/ml of bark extract (**A**) or 10 µg/ml of UF-concentrate (**B**) starting 2 h before infection or 0, 2, 4 or 6 h after infection. TCID50s were determined 24 h post infection. (**C**) Effect on HPV attachment. HaCaT cells preincubated for 1 h with the original (black bars) or tannin-free (grey bars) extracts or DMSO and infected for 15 min with 500 HPV pseudovirions per cell were washed five times and collected in SDS sample buffer for Western blotting. Cell-bound HPV16 particles were stained with anti-L1 antibody and relative band intensities to the β-actin band were quantified densitometrically. (**D**) Effect on HPV capsid disassembly. HaCaT cells were treated with 20 µg/ml of the extracts for 1 h before HPV 16 pseudovirion infection for 7 h followed by fixation and staining with mouse anti-L1-7 antibody. L1-7 recognizes an epitope located inside of the pseudovirion capsid accessible after uncoating. Fluorescence of L1-7-positive pixels was normalized to the cell nucleus signal (Hoechst staining) and expressed as % of untreated. n.d., not detectable or TCID50<1. * significant difference (p<0.05) as compared to “No treatment”.

To determine the step of the IAV life cycle affected by the bark extract and the UF-concentrate, A549 cells were infected with an MOI of 0.1 of pandemic H1N1, accompanied by treatment with 50 µg/ml of bark extract or 10 µg/ml of UF-concentrate 2 h before infection, at the time of infection or 2, 4 and 6 h after infection. The medium was replaced with drug-free medium 8 h post infection, which approximately corresponds to one IAV life cycle, to allow proliferation of intracellular virus to sufficient titers for another 24 h before titration. When drug treatment was started before or at the time of infection, no virus was detectable, while treatment starting at 2 h, 4 h or 6 h post infection induced slightly reduced but detectable virus titers as compared to the untreated control (Fig. 5AB). Therefore, an early step in the viral life cycle such as viral attachment or entry is inhibited. Treatment up to 6 h post infection also induced a decreased titer, suggesting that intermediary or late steps might be inhibited to a minor extent.

### Effect of Tannins and Pseudotannins on Viral Surface Protein Interactions

Since at least an early and a later step of the IAV life cycle seem to be inhibited and tannins are known to interact with proteins, we investigated the effect of the extracts and single compounds on the activity of the IAV surface proteins hemagglutinin and neuraminidase, involved in viral attachment and entry [69] or cleavage of nascent virions from the host cell [70]. In a hemagglutination inhibition assay, the UF-concentrate and the bark extract were the most active, while gallic acid, EGCG and hamamelitannin did not inhibit hemagglutination at concentrations up to >400 µg/ml (Table 3). Of note, the drugs also induced hemagglutination of virus-free erythrocytes at concentrations of at least >3.5-fold above HIC50 (data not shown), suggesting that they also interfere with cell surface proteins. After hide powder treatment of the active compounds, the hemagglutination inhibition disappeared at the tested concentrations, showing involvement of protein precipitating tannins in receptor binding inhibition. Interestingly, all tested extracts and compounds inhibited neuraminidase activity (Table 3), even in absence of tanning activity (gallic acid) or antiviral effect (hamamelitannin).

**Table 3:** Hemagglutination inhibition and neuraminidase inhibition.

	HIC50(µg/ml)	NIC50(µg/ml)	NIC50/HIC50
**Bark extract**	4.4	136.5	31.0
**UF-concentrate**	2.2	138.9	63.1
**UF-filtrate**	89.1	202.2	2.3
**Tannic acid**	14	125.3	9.0
**Gallic acid**	>400	106.6	<1
**EGCG**	>400	97.1	<1
**Hamamelitannin**	>400	147.8	<1

Half maximal hemagglutination inhibiting concentration (HIC50), half maximal neuraminidase inhibiting concentration (NIC50) of drug serial dilutions against pandemic H1N1 A/Luxembourg/46/2009 (Section 2.7).

Similarly, we investigated the effect of the bark extract, UF-fractions and single compounds on HPV 16 binding to its host cell receptor. 1 h of HaCaT cell preincubation with 50 µg/ml of the drugs before 15 min of incubation with 500 HPV pseudovirions per cell reduced HPV attachment to 49.7% or 32.9% for the bark extract and UF-concentrate, but not for UF-filtrate or single compounds (Fig. 5C). Tannin free extracts did not inhibit HPV attachment (Fig. 5C). 20 µg/ml of the bark extract and UF-concentrate also showed impaired HPV capsid disassembly/uncoating (11.7–12.9% of untreated control, Fig. 5D), probably a result of the reduced host cell binding.

### Effect of Preincubation of Virus or Cells with Hamamelis Extracts or Single Compounds

**Figure 6:**
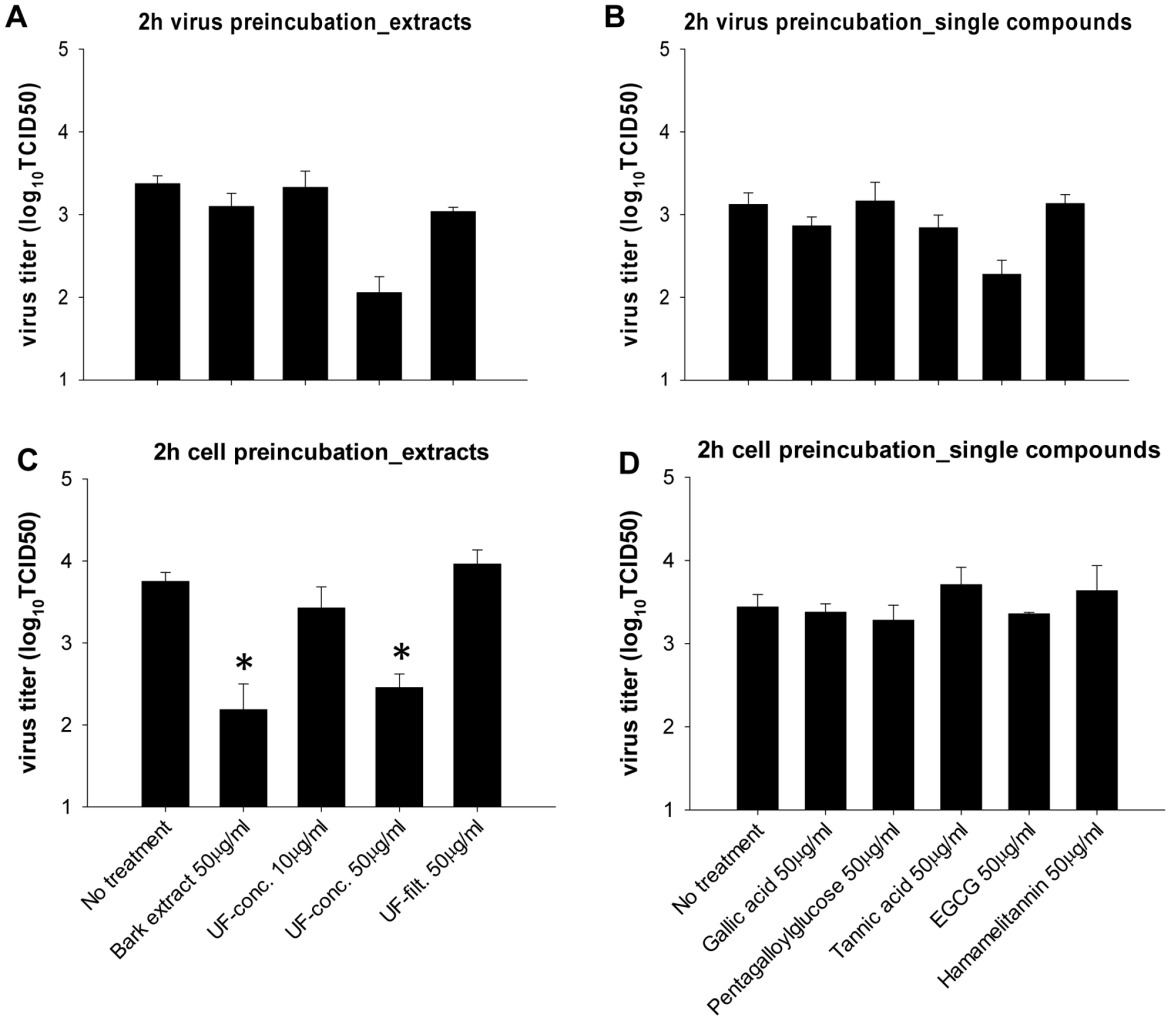
Effect of virus or cell preincubation with Hamamelis extracts or individual compounds. (**A–B**) Preincubation of pandemic H1N1 A/Lux/46/2009 for 2 h with virus growth medium (“no treatment”) or bark extract/UF-fractions (**A**) or individual compounds (**B**) before infection of A549 cells (MOI 0.1) and titration 24 h post infection (p.i.). (**C–D**) Preincubation of A549 cells for 2 h with virus growth medium (“no treatment”) or bark extract/UF-fractions (**C**) or single compounds (**D**) before three washes with PBS, infection with pandemic H1N1 (MOI 0.1) and titration 24 h p.i. All experiments were done in at least triplicates. * significant difference (p<0.05) as compared to “No treatment”.

After 2 h of preincubation at room temperature of pandemic H1N1 with Hamamelis bark full extract, the UF-fractions or single compounds, A549 cells were infected with an MOI of 0.1 (1/100 dilution resulting in negligible drug concentrations). Virus preincubation with 50 µg/ml of UF-concentrate or EGCG resulted in a roughly 20- or 7-fold lower viral titer, indicating an irreversible effect on IAV virus particles. The other extracts or single compounds did not notably influence IAV growth (Fig. 6AB).

Titers were significantly decreased (36- or 20-fold, respectively) when A549 cells were preincubated for 2 h with 50 µg/ml of full extract or UF-concentrate, washed three times with PBS and infected with an MOI of 0.1 of pandemic H1N1 for 24 h (Fig. 6CD), indicating an irreversible effect of these compounds on the host cells.

### Determination of Cytotoxicity or Unspecific Host Cell Receptor Inhibition

**Figure 7:**
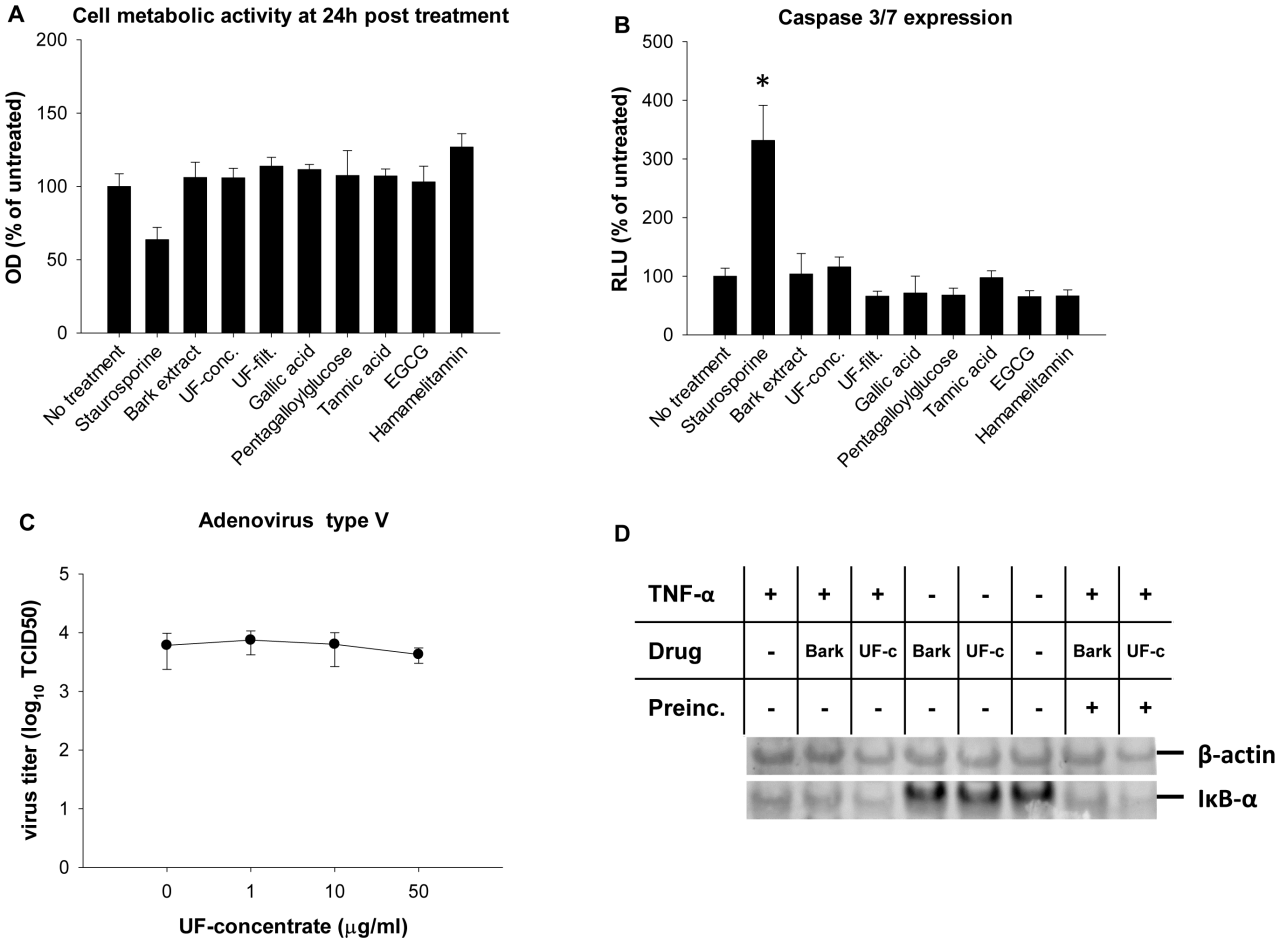
Determination of possible cytotoxicity or unspecific host cell receptor inhibition. (**A**) Cell metabolic activity after 24 h of incubation of A549 cells with DMSO (no treatment), 2.5 µM of staurosporine, 10 µg/ml of UF-concentrate or 50 µg/ml of the remaining drugs was determined in triplicates using XTT assay. Optical density (OD) was determined at 450 nm after background (650 nm) subtraction and expressed as % of the untreated samples. (**B**) Caspase 3/7 activity after 24 h of A549 cell incubation with DMSO (no treatment), 2.5 µM of staurosporine, 10 µg/ml of UF-concentrate or 50 µg/ml of the remaining drugs was assayed in at least triplicates using detection of a luminogenic caspase 3/7 cleavage product. (**C**) A549 cells were infected in triplicates with adenovirus type 5 (MOI of 0.05) and simultaneously treated with UF-concentrate. TCID50 was determined at 24 h post infection. (**D**) Interference of the drugs with cellular TNF-α signaling. A549 cells were preincubated for 30 or 0 minutes (“Preinc.”+or −, respectively) with 50 µg/ml of bark extract (“Bark”) or UF-concentrate (“UF-c”). Then, 0 or 30 ng/ml TNF-α were and 15 minutes later, total proteins were extracted. IκB-α and the loading control β-actin were detected on a Western blot using specific primary and Cy-5 and Cy-3 labeled secondary antibodies. * significantly elevated caspase expression (p<0.05) as compared to “No treatment”.

Antiviral drugs can mediate adverse effects by induction of cytotoxicity at antivirally active concentrations. In order to investigate this possibility, we determined CC50s using serial drug dilutions as described above (Sections 3.1., 2.2., 3.3., Fig. 2 AB & I, Fig. 3 AC, Table 1&2) and compared cell metabolic capacity of all extracts and compounds used in the study at 50 µg/ml or 10 µg/ml (UF-concentrate) by XTT assay. No important downregulation was found after 24 h of incubation, except for 2.5 µM staurosporine, a known apoptosis inducer used as a positive control (Fig. 7A). Apoptosis induction was monitored by luminescence quantification of a caspase 3/7 cleavage product. No significant caspase 3/7 upregulation was detected up to 50 µg/ml or 10 µg/ml (UF-concentrate) after 24 h of treatment, except for the positive control (Fig. 7B). In addition, A549 cells were infected by adenovirus type 5 (MOI 0.05) and incubated for 24 h in presence of different bark extract or UF-concentrate dilutions. The extracts did not affect adenoviral growth (Fig. 2H, Fig. 7C), showing that the cellular machinery (at least the part needed for adenoviral replication) was still functional. Thus, the extracts did not seem to exert cytotoxic or unspecific effects on the cell that would inhibit viral growth in general.

Since bark extract and UF-concentrate were shown to inhibit hemagglutinin interaction with its cellular receptor, we tested whether host cell surface proteins such as TNF- α were blocked unspecifically [61]. When TNF-α binds to its receptor, it induces the NFκB cascade and degradation of the NFκB inhibitor IκB-α. Treatment with bark extract or UF-concentrate starting 30 minutes before or at the time of A549 cell treatment with 30 ng/ml TNF-α did not influence IκB-α degradation (Fig. 7D). Thus, the bark extract and UF-concentrate do not inhibit activation of the TNF-α receptor as a model of an unrelated cellular receptor.

## Discussion

The study demonstrates the antiviral activity of Hamamelis bark extract against different IAV subtypes, systematically compares the activity of different tannin classes and structures and is the first report showing that a tannin-rich extract inhibits HPV or H7N9 subtype infection. Importantly, the antiviral efficacy was considerably increased in the UF-concentrate, an extract where high molecular weight condensed tannins were enriched by ultrafiltration. Interestingly, our results showed an increased benefit of the bark extract and especially the UF-concentrate, (SI of 94.7 and 325.5, respectively) compared to any of the individual hydrolysable (pseudo)tannins (SIs ranging from 15.2–40) or monomeric EGCG (SI 85). Since plant extracts normally contain different types of tannins, our observations are important for the development and improvement of plant-based antivirals.

The increased SI of the UF-concentrate above those of isolated compounds suggests a pronounced effect of the high molecular weight condensed tannins. For the bark extract, a synergistic effect of the different tannins in the extract could play a role. A similar effect has been demonstrated against some multiresistant nosocomial bacteria or *Streptococcus mutans* [71], [72]. Alternatively, the antiviral efficacy of the bark extract could be partially mediated by EGCG. However, it cannot be solely mediated by EGCG, since the bark extract and EGCG have approximately the same EC50, but the bark extract contains only 31% condensed tannins [47]. Also, the strong antiviral effect (SI 325.5) of the UF-concentrate is independent of EGCG, as it mainly contains tetrameric and longer condensed tannins. Of note, EGCG showed a roughly 2- to 6-fold higher SI than other single pseudotannins or tannic acid. The UF-concentrate showed by far the highest SI, although the 4.7-fold increase in anti-IAV efficacy was concomitant with a 1.4-fold increase in cytotoxicity, as compared to the bark full extract (Table 1). Since UF-concentration of a *Pelargonium sidoides* extract induced essentially no SI increase (84.4 to 86.3, data not shown) due to a concomitant increase of antiviral and cytotoxic effects, the benefit of fractionation by ultrafiltration (as well as the cut-off size of the ultrafiltration membrane) should be evaluated individually for every plant extract.

When single hydrolysable tannins were tested, their anti-IAV activity (on a molar basis) increased with the number of galloylations and cytotoxicity increased from pentagalloylglucose to highly galloylated tannic acid, resulting in the highest SI (SI = 40) for pentagalloylglucose. This effect of galloylation on antiviral efficacy has also been observed for herpes simplex virus [73], [74]. However, while tannic acid is nearly 12-fold more active than gallic acid on a molar basis, EC50s of both compounds are similar when expressed in µg/ml (1.3-fold difference, Table 2, in italics). Thus, the total number of galloyl residues determines the antiviral effect of hydrolysable tannins, irrespective of whether they are on the same or on different molecules.

We have shown that tannins are the active antiviral principle of Hamamelis-based extracts, as their depletion by hide powder abolishes antiviral activity (Fig. 4BC). Interestingly, tanning activity in *sensus stricto* (i.e. the ability to precipitate protein) is not essential for the anti-IAV activity as gallic acid does not precipitate hide protein but has antiviral activity (Fig. 4AC). Also, catechin monomers usually have only weak protein precipitating activity [21] but are well known for their antiviral efficacy [31], [36], [75], [76].

The bark extract and the UF-concentrate were shown to inhibit both an early and, to a lesser extent, a late step of the IAV life cycle (Fig. 5AB), and lost their anti-IAV activity when depleted of tannins (Fig. 4). While an effect of tannin-rich extracts on viral neuraminidase and hemagglutination has been observed before [31], [61], [77], the role of different (pseudo)tannins was not clear. Interestingly, the extracts and compounds rich in high molecular weight tannins and with a strong tanning activity upon incubation with hide powder (bark extract, UF-concentrate, tannic acid) inhibited hemagglutination at HIC50s as low as 4.4, 2.2 or 14 µg/ml, respectively. Their 9- to 63-fold higher NIC50s (Table 3) together with the strong inhibition of early steps in the IAV life cycle (Fig. 5AB) suggest that their effect on attachment contributes more to the antiviral activity than their effect on neuraminidase. In line with this, a significant correlation between EC50 and HIC50 (R^2^ = 0.997), but not NIC50 values, was observed for drugs inhibiting hemagglutination/neuraminidase activity in our assay. We have shown that gallic acid and EGCG, which do not inhibit hemagglutination, interfere with neuraminidase activity (Table 3). However, this inhibition is unlikely to play a role in the antiviral activity of gallic acid and EGCG, since also the antivirally inactive hamamelitannin inhibits neuraminidase at similar concentrations. Thus, it seems like the high molecular weight tannins tested in this study inhibit viral attachment by their tanning effects, while the antiviral activity of EGCG and gallic acid relies on different mechanisms. Previously proposed antiviral mechanisms for EGCG include inhibition of viral attachment [76], inhibition of endosomal acidification [78], membrane damage [79] or virus aggregation [75]. The anti-IAV mechanism of gallic acid remains to be determined. For herpes simplex virus, its virucidal activity was shown at concentrations below those that interfered with attachment and penetration [80].

Our experiments provide no indication whether the extracts inhibit/outcompete the viral surface protein (hemagglutinin or L1 for IAV or HPV, respectively), the host cell receptor (sialic acid or heparan sulfate proteoglycans), or both. Both have been suggested as targets for defined tannins or tannin-rich extracts: EGCG, theaflavin and persimmon extract bound to viral surface proteins and agglutinated IAV particles [66], [75]. Conversely, antiviral activities against herpes simplex virus of chebulagic acid and punicalagin, two high molecular weight tannins, were significantly reduced in cell lines deficient in heparan sulfate [81].

It was shown earlier that HPVs of various types use heparan sulfate proteoglycans (HSPGs) for binding to the host cell surface. Complex formation with additional HSPG molecules induces conformational changes of the virus capsid [82] allowing transfer to the second receptor complex [15], internalization into the infectious entry pathway, and uncoating/disassembly in the endocytic compartment (for review see [83], [84]). By interfering with this virus/HSPG interaction, tannins seem to affect not only primary attachment but also further steps required for capsid disassembly which would lead to the observed reduction of virus cell binding and nearly loss of capsid disassembly and infection.

In our preincubation experiments (Fig. 6A, C), we observed an irreversible effect of high molecular weight tannins of the UF-concentrate on the virus particle as well as on the host cell at 50 µg/ml. In contrast, the UF-filtrate (rich in low molecular weight tannins) and single hydrolysable tannins seem to have either no or only a reversible effect in this assay. In line with this observation, the protein binding efficiency of tannins increases with molecular size [26] and the number of galloyl residues [85], [86], suggesting a tighter binding of high molecular weight tannins to target proteins. In addition to interfering with surface proteins, a virucidal activity (e.g. by membrane damage) has been proposed for EGCG [33], [79] and could explain the 7-fold decrease in titer after virus preincubation with EGCG. Our preincubation experiment did not allow discriminating between a virucidal activity and an irreversible inhibition of viral proteins. Interestingly, cell but not virus preincubation with the bark extract lead to reduced viral titers (Fig. 6A, C), which may be due to a higher affinity of bark extract tannins to cellular over viral surface proteins.

Titers >10^2^ of pandemic H1N1 virus were still detected (Fig. 6A, C) upon preincubation of either the virus or the cell with 50 µg/ml of UF-concentrate, while viral growth was minimal when only 10 µg/ml of UF-concentrate were added at the time of infection (Fig. 3A). This suggests that in addition to irreversible effects, reversible effects play a role, e.g. reversible inhibition of surface proteins or surface-independent effects. For instance, tannins stimulated innate immunity in infected PBMCs in the case of dengue virus [87].

While inhibiting both IAV and HPV, the effect of the bark extract and UF-concentrate was nevertheless not unspecific, since for example adenovirus was not inhibited up to >50 µg/ml. Also, the hemagglutination assay and Western blot showed that the bark extract and UF-concentrate inhibited viral attachment to the host cell both for IAV (Table 3) and HPV (Fig. 5C), but not the TNF-α receptor activity (Fig. 7D), demonstrating some level of specificity.

In this study, we have described for the first time the anti-influenza and anti-HPV activity of *Hamamelis virginiana* L. Importantly, we directly compared the anti-IAV effects of full extracts, fractions enriched in tannins of different molecular weights and single defined tannins or pseudotannins. We provided further insight into the structural basis of the anti-IAV activity of tannins and into the steps of the viral life cycle that are affected. We also showed interesting structure-related differences in receptor binding inhibition capacities and pointed out the probably low contribution of neuraminidase inhibition to the antiviral activity. Finally, we identified a highly potent fraction against both IAV and HPV that was enriched in high molecular weight tannins by simple and reproducible ultrafiltration.
